# Suicidal behaviours among adolescents from 90 countries: a pooled analysis of the global school-based student health survey

**DOI:** 10.1186/s12889-020-09209-z

**Published:** 2020-08-10

**Authors:** Susan C. Campisi, Bianca Carducci, Nadia Akseer, Clare Zasowski, Peter Szatmari, Zulfiqar A. Bhutta

**Affiliations:** 1 Centre for Global Child Health, Hospital for Sick Children, 686 Bay Street, 11th Floor, Suite 11.9805, Toronto, ON M5G 0A4 Canada; 2 Department of Nutritional Sciences, Faculty of Medicine, University of Toronto, Medical Sciences Building, 1 King’s Circle College, Toronto, Ontario M5S 1A8 Canada; 3 School of Nutrition, Faculty of Community Service, Ryerson University, Kerr Hall South, Room KHS-349; 50 Gould Street, Toronto, Ontario M5B 1X8 Canada; 4 Department of Psychiatry, Hospital for Sick Children, 555 University Avenue, Burton Wing, Toronto, Ontario M5G 1X8 Canada; 5 Department of Psychiatry, University of Toronto, 250 College Street, 8th floor, Toronto, Ontario M5T 1R8 Canada; 6 Centre for Addiction, and Mental Health, Cundill Centre for Child and Youth Depression, 80 Workman Way, Toronto, Ontario M6J 1H4 Canada; 7 Centre of Excellence in Women, and Child Health, Aga Khan University, Stadium Road, PO Box 3500, Karachi, 74800 Pakistan; 8 Dalla Lana School of Public Health University of Toronto, Health Sciences Building, 155 College Street, 6th floor, Toronto, Ontario M5T 3M7 Canada

**Keywords:** Adolescent mental health, Prevalence, Risk indicators, Suicidal ideation, Suicide attempt

## Abstract

**Background:**

Understanding the burden and determinants of suicide during adolescence is key to achieving global health goals. We examined the prevalence and determinants of self-reported suicidal ideation and attempts among younger (13–15 years) and older adolescents (16–17 years).

**Methods:**

Pooled prevalence estimates with 95% confidence interval, were calculated for suicide ideation and attempts for 118 surveys from 90 countries that administered the Global School-based Student Health Survey (GSHS) to adolescents (13–17 years of age) from 2003 to 2017. Indicators (including individual and social factors) associated with suicidal ideation and attempts were determined from multivariable linear regressions on key outcomes.

**Results:**

The prevalence of suicidal ideation representing 397,299 adolescents (51.3% female) was significantly higher among girls than boys whereas attempts did not differ by age or sex. Being bullied, or having no close friends was associated with suicidal ideation among girls 13–15 years and 16–17 years, respectively. Among all boys, being in a fight and having no close friends was associated with suicidal ideation with the addition of serious injury for boys 13–15 years. Common to all younger adolescents was an association of suicide attempt with being bullied and having had a serious injury. Among young boys, having no close friends was an additional indicator for suicide attempt. Having no close friends was associated with suicide attempt in older adolescents with the addition to being bullied in older girls and serious injury in older boys.

**Conclusions:**

Building positive social relationships with peers and avoiding serious injury appear key to suicide prevention strategies for vulnerable adolescents. Targeted programs by age group and sex for such indicators could improve mental health during adolescence in low and middle-income countries, given the diverse risk profiles for suicidal ideation and attempts.

## Background

Death by intentional self-directed injury, suicide, is an indicator for Sustainable Development Goal 3.4.2 [1]. Suicide amid young people (15–29 years) accounts for one-third of all suicides globally and is the second leading cause of death in this age group [2]. Global estimates from the first decade (2000–2009), for suicide mortality in early adolescents (10–14 year of age) is 1.52 per 100,000 for boys and 0.94 per 100,000 for girls which jump to 10 per 100,000 during late adolescence (15–19 year of age) [3, 4]. Furthermore, a recent meta-analysis of 24 studies which assessed risk and protective factors of suicidal behaviours in adolescents and young adults (14–26 years) suggested that females presented with a higher risk of suicide attempt (Odds Ratio (OR): 1.96, 95% Confidence Interval (CI): 1.54–2.50), as compared to males [5]. Suicidal behaviour includes ideation (thinking about killing oneself), planning suicide, attempting suicide and completed suicide [6]. Examination of determinants of suicidal behaviour within this vulnerable age group is critical to its prevention and early intervention.

The need to recognize differences in suicidal behaviour between younger and older adolescents stems from the observed sex-paradox of suicidal behaviour which becomes true at about 15 years of age and indicates that suicidal ideation, planning and attempts are higher among females and ‘completed’ suicide is higher in males [7–9]. Thus, older adolescents may have different determinants associated with suicidal behaviours and in many low- and middle- income countries (LMIC) are more likely to be out of school [5, 10]. Additionally, strong evidence exists for different risk indicators for suicidal ideation and attempts by sex with many studies reporting peak suicidal behaviour around 15 years-of-age with the exception of Korean girls, where suicide attempts peak at 13 years of age [11, 12].

Elucidating the pathway towards ‘completed’ suicide among early adolescents is hindered by a low prevalence rate and the heterogeneity of empirical evidence. It is believed that suicidal behaviour is rooted in nonlinear interactions with multiple risk indicators broadly including environmental, biological and psychological factors. Furthermore, many of these factors are bidirectionally impacted by poor access to health services in many LMIC [13–20].

Several groups have explored suicide behaviour in LMIC using data from the Global School-based Student Health Survey (GSHS). Uddin et al. reported that the African region had the highest prevalence of suicidal ideation (20.4%) and suicide planning (23.7%), while the western Pacific region had the highest prevalence of suicide attempts (20.5%) among adolescents (13–17 years) in 59 LMIC using GSHS data from 2003 to 2015 [21]. This study, however, did not examine any associations with risk or protective factors for suicidal behaviour. A study by McKinnon et al. identified loneliness, alcohol use and bullying with a greater risk ratio among 13–17-year-old adolescents as determinants of suicide ideation and attempt in 32 LMIC using early data from the GSHS [22]. Both studies excluded GSHS high-income countries (HIC) and did not disaggregate early adolescents from late adolescents despite their vastly different suicide mortality rates. Most recently, a study by Koyanagi using GSHS data (2009–2015) found that amongst adolescents (12–15 years) from 48 countries (39 LMIC), being bullied for at least 1 day in the past 30 days was associated with a more than 2-fold higher odds for overall suicide attempt [23]. While this study limited the data to younger adolescents, it severely restricted the available GSHS data by date without explanation and did not include other potential risk indicators.

Robust multi-national data to measure risk and protective factors towards suicide behaviour among early adolescents compared to older adolescents, can provide important evidence leading to targeted support and services for these two vulnerable age groups. Clearly, there is an increased interest in suicidal behaviour research among adolescents living in LMIC, yet previously published studies using GSHS data have not examined differences between early and late adolescents by sex and fail to use all available GSHS datasets.

With the aim of including the most up-to-date GSHS results and a comparative analysis between early and late adolescence, we examine GSHS data (2003–2017) among 13–17-year-old respondents, stratified by age and sex, to determine the association of self-reported risk indicators with suicidal ideation and attempts.

## Methods

### Data source

We used data from the GSHS implemented in 90 countries. The GSHS is a cross-sectional surveillance survey that aims to collect data among adolescents. The survey is designed to determine the prevalence of internationally comparable health behaviours and risk indicators among adolescents attending school. Country-specific surveys are composed of a select core and core-expanded questions, which can be completed during one school class period. Core questions common to all phases maintained the exact wording in the questions and responses. There were three phases of the GSHS: phase one (2003–2008); phase two (2009–2012) and phase three (2013 to 2019). Detailed methodology of the GSHS is previously reported [24]. As some countries participated in more than one phase of the survey, our final dataset included 118 surveys. Only regional or city data were available for five countries (Zimbabwe, Chile, China, Ecuador and Palestine); all other estimates are nationally representative. Countries were classified according to current the World Bank (WB) Income Groups and the WHO regions. All GSHS surveys obtain approval in their country by a national government agency and an institutional ethics board or committee. Parental consent and participant assent are also obtained. Individual country ranking by sex and age group for mean prevalence estimates for study outcomes by WB Income Group and WHO region are listed in Supplementary Figs. 1–2.

The focus of the current analysis divides adolescents into younger (13–15 years of age) and older (16–17 years of age) groups. These age groups were selected as they are consistent with the publicly reported question response prevalence, which is weighted for the survey design to allow for comparability across countries by accounting for the probability of (a) selection of schools and classrooms, (b) non-responding schools and students and (c) distribution of the population by grade and sex [24].

### Outcomes and covariates

One outcome variable – suicidal ideation – was included in all three phases of the survey whereas the second outcome variable - suicide attempt – was only included in phases two and three. Covariates that could potentially explain our outcomes were chosen from core questions using identical wording in at least two phases of the GSHS, from the following modules – mental health, physical activity, protective, violence and unintentional injury (Table 1). All covariates were included in all three phases of the survey except for ‘parental understanding’ which was included in phases two and three.

**Table 1:** Global School-Based Health Survey (GSHS) Questions used in the current analysis of adolescent suicidal behaviours

Variable	Core Question	Dichotomous response	Item Rationale for Current Analysis
Outcomes			
Suicide Behaviours			
Suicidal ideation	During the past 12 months, did you ever seriously consider attempting suicide?	Percentage of students who ever seriously considered attempting suicide during the 12 months before the survey	Prior suicidal ideation is a risk indicator for attempts [20]
Suicide Attempt	During the past 12 months, how many times did you actually attempt suicide?	Percentage of students who attempted suicide one or more times during the 12 months before the survey	Prior suicide attempt is a significant risk indicator for future suicide fatality [20]
Covariates			
*Distal Factors*			
Parental understanding	During the past 30 days, how often did your parents or guardians understand your problems and worries?	Percentage of students who reported that their parents or guardians most of the time or always understood their problems and worries during the 30 days before the survey	Parental involvement is associated with lower levels of depression and suicidal ideation [14]
No Close friends	How many close friends do you have?	Percentage of students who did not have any close friends	Decreased interaction with friends worsens social skills and undermines the ability to cope with stressful events [14]
Being Bullied^a^	During the past 30 days, on how many days were you bullied?	Percentage of students who were bullied on one or more days during the 30 days before the survey	Victimization due to bullying is connected to many negative outcomes including depression, suicidal ideation, self-injury and suicide attempts [16]
Physical fight	During the past 12 months, how many times were you in a physical fight?	Percentage of students who were in a physical fight one or more times during the 12 months before the survey	Physical fighting can be linked to difficult behaviours and serious injury-related health outcomes [15]
PE Class	During the school year, on how many days did you go to physical education (PE) class each week?	Percentage of students who went to physical education (PE) class on three or more days each week during the school year	School PE classes can increase adolescent participation in physical activity, increase school performance and develop skills and attitudes for lifelong physical activity [13]
Serious Injury ^b^	During the past 12 months, how many times were you seriously injured?	Percentage of students who were seriously injured one or more times during the 12 months before the survey	Unintentional injuries can lead to disabilities, depression and risky behaviours [17]
Physical Activity	During the past 7 days, on how many days were you physically active for a total of at least 60 min per day?	Percentage of students who were physically active for a total of at least 60 min per day on five or more days during the past seven days	Regular physical activity promotes healthy physical health and well-being while reducing depression and anxiety [13]

^a^Bullying is defined when “a student or group of students say or do bad and unpleasant things to another student” or “when a student is teased a lot in an unpleasant way” or “when a student is left out of things on purpose”. The survey questionnaire specifies that “it is not bullying when two students of about the same strength or power argue or fight or when teasing is done in a friendly and fun way”

^b^Serious Injury is defined as when, “you miss at least one full day of usual activities (such as school, sports, or a job) or requires treatment by a doctor or nurse”

### Statistical analysis

Pooled prevalence estimates with 95% confidence interval (95% CI), were calculated by *random*-*effects meta*-*analyses using the* DerSimonian and Laird inverse variance method by sex for adolescent age groups (13–15 years, 16–17 years) [25]. We determined pooled estimates to be significantly different if the 95% confidence intervals did not overlap.

The level of heterogeneity was assessed using *I*^2^ among country surveys; *I*^2^ was greater than 80% suggesting substantial heterogeneity among countries for all variables by sex and age groups. To test differences between HIC and LMIC, pooled prevalence estimates with 95% confidence interval (95% CI) were calculated by *random*-*effects meta*-*analyses* as outlined above by income group for sex and adolescent age groups (13–15 years, 16–17 years). We found no meaningful differences in pooled estimates according to country income group (Supplementary Figs. 3–6).

Linear multiple regression was used to test if protective or risk indicators were significantly associated with suicide attempts or ideation by sex for each age group. Each estimated β coefficient was interpreted in proper units and tested for significance by performing an analysis of variance (ANOVA). Variables significant at a liberal *p*-value of less than 0.2 in the bivariate models were considered in the development of a multivariable model using backwards stepwise regression selection. Multivariable models were adjusted for sex, year of survey, WB Income Group and the survey sample size to control for varying populations and contexts. Multicollinearity among selected covariates was assessed using variance inflation factors; a cut-off of three was considered suspect for collinearity. We computed a standardized β coefficient with ordinary least squares regressions. The effect size explained by the model (R^2^) and the coefficient intervals for the covariates were computed. To assess the significance of the linear regression model, the F-statistic was computed from the ANOVA and interpreted at the α = 0.05 significance level. To validate the model, overfitting was assessed using Bradley Efron’s .632 bootstrap resampling technique. Bootstrap is a non-parametric method that computes the inferential estimate of interest without making assumptions about the distribution [26]. Instead, bootstrap treated the actual sample as the source population and repeatedly drew samples from 63.2% of the actual sample to compute a nearly unbiased estimate of the optimism in the fitted model – accounting for variations caused by the modelling strategy [26].

Two sensitivity analyses were conducted to verify the use of the entire dataset thereby increasing the robustness of the data. The first sensitivity analysis assessed the use of more than one phase per country. Since some countries reported more than one GSHS phase for adolescents aged 13–15 years, all estimates were restricted to only those countries that participated in all survey phases and another for those countries that participated in 2 phases. We found no meaningful differences in mean estimates based on this restricted set of countries allowing for the use of multiple phases datasets per country in the regression analysis (Supplementary Figs. 7 and 8). Second, prevalence estimates for 16–17-year-old adolescents were only available for phase two and three and no countries reported more than one phase of the older adolescent data. For this sensitivity analysis, the dataset was limited to the younger age group. Mean estimates were calculated for all variables and stratified according to those with and without the older age group. No meaningful differences were observed, allowing for the entire dataset thereby increasing the robustness of the data (Supplementary Fig. 9). We considered *p*-values less than 0.05 as statistically significant. The *random*-*effects meta*-*analyses* were conducted using RevMan 5.3 all other analysis was executed using R.3.5.1 statistical software.

## Results

### Income-level analysis

This analysis represents 310,055 (51.7% female) students aged 13–15 years and 87,244 (49.6% female) aged 16–17 years. Of the 118 surveys used in the analysis, 26 (22.0%) were from HIC, 42 (35.6%) were from upper-middle-income countries, 34 (28.8%) from lower-middle-income countries and 13 (11.0%) from low-income countries, 3 (2.5%) came from unclassified countries. Regionally, 19 (16.1%) were from the African Region, 38 (32.2%) were from the Americas, 19 (16.1%) from Eastern Mediterranean, 2 (1.7%) from European Region, 14 (11.9%) from South-East Asia and 26 (22.0%) from the Western Pacific Region.

### Age and sex - level analysis

**Fig. 1:**
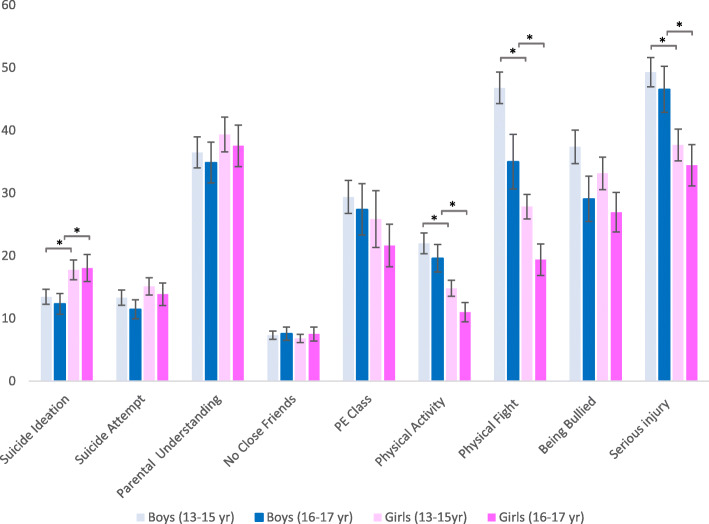
Pooled prevalence estimates of suicide behaviours, risk and protective factors by sex and age group. Legend. *indicates a *p-*value< 0.001

Few differences in the pooled prevalence’s were significant. Here, we highlight some of the trends from Fig. 1. The pooled prevalence of suicide ideation was lower for boys than girls, with little difference between age groups of the same sex. Conversely, the pooled prevalence of suicide attempts did not differ greatly by sex or age group. The pooled prevalence of parental understanding and having no close friends were similar by age group and sex. Physical activity, attending PE class, being in a physical fight, having a serious injury and begin bullied decreased with age. Additionally, physical activity, being in a physical fight, and having a serious injury were significantly less prevalent among girls.

### Country-level analysis

Suicide ideation was the lowest for girls and boys 13–15 years living in Myanmar (survey year 2007) at 0.8 ± 0.5% and 0.7 ± 0.7% and for 16–17 years girls and boys living in Laos (survey year 2015) at 1.6% (95% CI:1.7–3.9) and 3.9% (95% CI: 2.6–6.8) respectively. The highest prevalence of suicide ideation among boys 13–15 years was in Samoa (survey year 2011) at 37.1% (95% CI: 33.8–40.5) among boys 16–17 was 22.8% (95% CI: 18.0–28.4) in Liberia (survey year 2017). In Kiribati (survey year 2011) girls, 13–15 years at 36.2% (95% CI: 31.9–41.7) and in Wallis and Futuna (survey year 2015) girls 16–17 at 35.9% (95% CI: 29.8–42.5) had the highest prevalence for suicide ideation.

The lowest prevalence for reported suicide attempts was among 13–15-year-olds living in Suriname (survey year 2016) at 4.2% (95% CI: 2.1–8.2) for boys and living in Indonesia (2015) (3.6% (95% CI: 2.9–4.5) for girls. Among 16–17-year-old adolescents the lowest prevalence for girls was among those living in Indonesia (survey year 2015) at 2.7% (95% CI: 1.7–4.1) and for boys was among those living in Laos (survey year 2015) at 3.8% (95% CI: 2.2–6.3). The highest prevalence of suicide attempts among 13–15-year-old boys and girls was for those living in Samoa (survey year 2011) at 67.2% (95% CI: 59.2–74.3) and (53.7% (95% CI: 45.1–62.1) respectively. Among 16–17-year-old boys, the highest prevalence for suicide attempts was 33.6% (95% CI: 28.3–39.4) for those living in Liberia (survey year 2017) and girls living in Ghana (survey year 2012) at 35.2% (95% CI: 24.3–47.9).

### Factors associated with suicidal behaviour

The bivariate and multivariable assessments of factors related to suicidal ideation for those 13–15 years are presented in Table 2. Factors associated with suicidal ideation varied by sex. For girls, being bullied (β = 0.4, *p* < 0.001) remained a significant predictor in the multivariable suicidal ideation model, where one covariate explained 23% of the variance (R^2^ = 0.23, F (5,107) = 6.3, *p* < 0.001). Whereas for boys, having a serious injury (β =0.3, *p* < 0.001), no close friends (β =0.3, *p* < 0.001) and being in a physical fight (β =0.3, *p* < 0.01) remained statistically significant in the multivariable suicidal ideation model, where these three factors explained 52% of the variance in suicidal ideation (R^2^ = 0.5, F (7,103) =16.8, *p* < 0.001). Multivariable regression results detailing the covariates that significantly associated with suicide attempt for those 13–15 years are presented in Table 3. In sex-stratified analyses, being bullied (β = 0.3, *p* < 0.01) and having had a serious injury (β = 0.4, *p* < 0.001) remained a significant factor for girls in the multivariable suicide attempt model, where these two covariates explained 52% of the variance (R^2^ = 0.5, F (6, 69) =12.3, *p* < 0.001). Whereas for boys, being seriously injured (β =0.4, *p* < 0.001), being bullied (β =0.3, *p* < 0.001) and having no friends (β =0.3, *p* < 0.001) all remained significant predictors in the multivariable suicide attempt model, where these three covariates explained 69%of the variance (R^2^ = 0.7, F (7, 67) =20.8, *p* < 0.001).

**Table 2:** Factors associated with suicidal ideation by sex, 13–15 years of age

	Girls aged 13–15 years							Boys aged 13–15 years						
	Bivariate				Multi-variable (*n* = 108) (adjusted for survey sample size, survey year and country income level)			Bivariate				Multi-variable (*n* = 104) (adjusted for survey sample size, survey year and country income level)		
Variable	*n*	β	95% CI	*p-*value	β	95% CI	*p-*value	*n*	β	95% CI	*p-*value	β	95% CI	*p-*value
Survey Sample Size	116	0.07	−0.001; − 0.0001	0.46				115	−0.06	−0.001; − 0.0002	0.54			
Survey Year	116	−0.13	−0.54; 0.09	0.15				115	−0.15	−0.50; 0.05	0.11			
Income level	116							115						
HIC		Ref							Ref					
LMIC		−0.18	−2.52; 9.07	0.27					0.17	−1.15; 4.18	0.35			
Parental Understanding	76	−0.13	−0.20; 0.06	0.26				71	−0.25	−0.26; 0.15	0.03			
Serious Injury	111	0.35	0.09; 0.27	< 0.001				112	0.57	0.20; 0.36	< 0.001	0.33	0.34; 0.84	< 0.001
Physical Fight	111	0.30	0.04; 0.28	0.01				112	0.48	0.15; 0.48	< 0.001	0.29	0.06; 0.21	< 0.001
No friends	115	0.13	−0.10; 0.55	0.17				115	0.47	0.52; 1.08	< 0.001	0.34	0.34; 0.08	< 0.001
Being Bullied	112	0.31	0.07; 0.26	< 0.001	0.37	0.10; 0.29	< 0.001	113	0.49	0.15; 0.30	< 0.001			
Physical Activity	108	0.19	0.003;0.36	0.05				101	−0.02	−0.15; 0.12	0.84			
Phys.Ed. Class	74	0.02	−0.13; 0.16	0.90				74	0.24	0.01; 0.26	0.04			
					*R*^*2*^		0.23					*R*^*2*^		0.52
					*R*^*2*^*(adjusted)*		0.19					*R*^*2*^*(adjusted)*		0.49
					*F (5,107)*	6.27	< 0.001					*F (7,103)*	16.18	< 0.001

Abbreviation: *β* beta

**Table 3:** Factors associated with suicide attempt by sex, 13–15 years of age

	Girls aged 13–15 years							Boys aged 13–15 years						
	Bivariate				Multi-variable (*n* = 70) (adjusted for survey sample size, survey year and country income level)			Bivariate				Multi-variable (*n* = 68) (adjusted for survey sample size, survey year and country income level)		
Variable	*n*	β	95% CI	*P-*value	β	95% CI	*p-*value	*n*	β	95% CI	*P-*value	β	95% CI	*p-*value
Survey Sample Size	77	0.22	− 0.001; 0.002	0.05				117	0.12	−0.001; 0.002	0.31			
Survey Year	77	−0.21	−1.26; 0.03	0.06				78	−0.13	−1.26; 0.32	0.24			
Income level	76							76						
HIC		Ref							Ref					
LMIC		0.04	−3.61; 4.96	0.75					0.15	−1.254; 17.152	0.22			
Parental Understanding	70	−0.16	−0.25; 0.06	0.20				71	−0.30	− 0.42; − 0.06	0.01			
Injury	75	0.60	0.23; 0.44	< 0.001	0.41	0.09; 0.36	0.001	76	0.72	0.42; 0.66	< 0.001	0.41	0.15; 0.47	< 0.001
Fight	75	0.42	0.14; 0.43	< 0.001				76	0.46	0.17; 0.45	< 0.001			
No friends	76	0.27	0.08; 0.89	0.02				76	0.47	0.66; 1.66	< 0.001	0.28	0.32; 1.05	< 0.001
Bullying	75	0.56	0.20; 0.41	< 0.001	0.29	0.02; 0.29	0.02	76	0.66	0.33; 0.57	< 0.001	0.32	0.07; 0.38	0.005
Physical Activity	73	0.22	10.01; 0.42	0.06				74	−0.03	−0.26; 0.19	0.77			
PE Class	70	0.18	−0.04; 0.30	0.13				71	0.15	−0.07; 0.34	0.20			
					*R*^*2*^		0.52					*R*^*2*^		0.69
					*R*^*2*^*(adjusted)*		0.474					*R*^*2*^*(adjusted)*		0.65
					*F (6,69)*	12.28	< 0.001					*F (7,67)*	20.82	< 0.001

Abbreviation: *β* beta

The bivariate and multivariable assessments of factors related to suicidal ideation for those 16–17 years are presented in Table 4. Factors associated with suicidal ideation varied by sex. For girls, only having no close friends (β = 0.4, *p* < 0.01) remained a significant predictor in the multivariable suicidal ideation model, where one covariate explained 32% of the variance (R^2^ = 0.3, F (5, 38) =3.6, *p* = 0.01). Whereas for boys, being in a physical fight (β =0.6, *p* < 0.001) and having no close friends (β =0.5, *p* < 0.001) remained statistically significant in the multivariable suicidal ideation model, where these two factors explained 55%of the variance in suicidal ideation (R^2^ = 0.5, F (6, 38) =7.7, *p* < 0.001). Multivariable regression results detailing the covariates that significantly associated with suicide attempt for those 16–17 years are presented in Table 5. In sex-stratified analyses, being bullied (β = 0.5, *p* < 0.001) and having no close friends (β = 0.4, *p* < 0.001) remained a significant factor for girls in the multivariable suicide attempt model, where these two covariates explained 62% of the variance (R^2^ = 0.6, F (6, 36) =9.9, *p* < 0.001). Whereas for boys, being seriously injured (β =0.6, *p* < 0.001), having no close friends (β =0.4, *p* < 0.01) a remained significant predictor in the multivariable suicide attempt model, where these three covariates explained 56%of the variance (R^2^ = 0.6, F (6, 36) =7.7, *p* < 0.001).

**Table 4:** Factors associated with suicidal ideation by sex, 16–17 years of age

	Girls aged 16–17 years							Boys aged 16–17 years						
	Bivariate				Multi-variable (*n* = 39) (adjusted for survey sample size, survey year and country income level)			Bivariate				Multi-variable (*n =* 39) (adjusted for survey sample size, survey year and country income level)		
Variable	*n*	β	95% CI	*p-*value	β	95% CI	*p-*value	*n*	β	95% CI	*p-*value	β	95% CI	*p-*value
Survey Sample Size	43	0.07	−0.001;0.001	0.64				44	−0.03	−0.00; 0.001	0.82			
Survey Year	43	0.12	−1.01;2.23	0.43				44	−0.03	−1.12; 1.02	0.86			
Income level	42							43						
HIC		Ref												
LMIC		−0.22	−9.59;1.46	0.15					0.06	−5.80; 8.31	0.84			
Parental Understanding	40	−0.09	−0.27;0.15	0.57				41	−0.16	−0.23; 0.07	0.30			
Serious Injury	43	0.12	−0.10; 0.23	0.43				42	0.52	0.11; 0.35	< 0.001			
Physical Fight	43	0.10	−0.20; 0.37	0.52				43	0.50	0.09; 0.32	< 0.001	0.59	0.15; 0.34	< 0.001
No friends	43	0.31	0.02; 0.94	0.04	0.39	0.18; 1.05	0.006	44	0.36	0.03; 0.27	0.002	0.53	0.39; 0.98	< 0.001
Being Bullied	43	0.14	−0.11; 0.27	0.38				43	0.41	0.05; 0.29	0.01			
Physical Activity	41	0.37	0.11; 0.97	0.02				42	−0.13	−0.28; 0.11	0.39			
Phys.Ed. Class	39	−0.01	− 0.22, 0.21	0.97				40	−0.01	− 0.13; 0.12	0.94			
					*R*^*2*^		0.32					*R*^*2*^		0.55
					*R*^*2*^*(adjusted)*		0.23					*R*^*2*^*(adjusted)*		0.48
					*F (5,38)*	3.59	*P =* 0.01					*F (6, 38)*	7.74	*P <* 0.001

Abbreviation: *β* beta

**Table 5:** Factors associated with suicide attempt by sex, 16–17 years of age

	Girls aged 16–17 years							Boys aged 16–17 years						
	Bivariate				Multi-variable (*n* = 37) (adjusted for survey sample size, survey year and country income level)			Bivariate				Multi-variable (*n =* 37) (adjusted for survey sample size, survey year and country income level)		
Variable	*n*	β	95% CI	*P-*value	β	95% CI	*p-*value	*n*	β	95% CI	*P-*value	β	95% CI	*p-*value
Survey Sample Size	42	0.05	−0.001; 0.001	0.77				43	0.04	−0.00; 0.02	0.77			
Survey Year	42	0.07	−1.16; 1.82	0.66				43	0.05	−1.21; 1.67	0.75			
Income level	41							43						
HIC		Ref							Ref					
LMIC		−0.02	−5.8; 5.30	0.93					0.12	−6.19; 11.56	0.51			
Parental Understanding	39	−0.03	− 0.21; 0.17	0.83				40	−0.13	−0.28; 0.12	0.43			
Injury	42	0.55	0.14; 0.39	< 0.001				42	0.63	0.22; 0.50	< 0.001	0.57	0.19; 0.45	< 0.001
Fight	42	0.50	0.18; 0.62	< 0.001				43	0.28	−0.01; 0.30	0.07			
No friends	42	0.63	0.51; 1.17	< 0.001	0.44	0.27; 0.91	< 0.001	43	0.48	0.34; 1.22	< 0.001	0.39	0.27; 1.03	< 0.001
Bullying	42	0.64	0.21; 0.48	< 0.001	0.51	0.15; 0.40	< 0.001	43	0.48	0.11; 0.39	< 0.001			
Physical Activity	40	0.12	−0.24; 0.56	0.42				40	−0.24	−0.44; 0.05	0.12			
PE Class	38	0.23	−0.06; 0.32	0.17				39	0.03	−0.15; 0.18	0.84			
					*R*^*2*^		0.62					*R*^*2*^		0.56
					*R*^*2*^*(adjusted)*		0.56					*R*^*2*^*(adjusted)*		0.49
					*F (6,36)*	9.88	< 0.001					*F (6,36)*	7.73	< 0.001

Abbreviation: *β* beta

## Discussion

Across all countries included in this analysis, the prevalence of suicidal ideation among adolescents aged 13–17 years is strikingly higher among girls than boys with very little differences between younger and older adolescents. However, suicide attempt did not differ by age group or sex. From our multivariable model, significant indicators associated with suicidal ideation or attempt included being bullied, having no close friends, being in a physical fight and/or a having had a serious injury. Not surprisingly, indicators related to parental connection or country income status failed to be significant, whereas those related to peer connection or isolation were significantly associated with suicide ideation and/or attempts. The convergence of bullying, aggression (fighting) and no friends reflects a problem in social relationships during the entire phase of adolescence, with little difference between HIC and LMIC.

Over the last 15 years, GSHS has collected information on the health behaviours in adolescents. Comparable tools, such as the Health Behavior in School-aged Children (HBSC), Youth Risk Behavior Surveillance System (YRBSS) and Korean Youth Risk Behavior Web-based Survey (KYRBS) have generated knowledge on suicidal ideation and suicide attempts in adolescents in predominantly developed countries [27–29]. The sex paradox observed in large epidemiological surveys conducted in high-income countries, including the YRBSS and KYRBS, where adolescent females more often plan and attempt suicide, while adolescent males more often complete suicide was not apparent in the GSHS data [28, 29].

Respondents of the Samoan GSHS (2011) experienced a disproportionately higher burden of suicidal ideation and attempt compared to other countries, particularly among boys. (Supplementary Figs. 1 and 2). This finding is consistent with historical data on youth suicide in Samoa. Countries in the Pacific Region, including Samoa, experienced epidemic youth suicide rates between 1960 to 1995 with a peak in 1980 [30]. High suicide rates in Samoa continued until 2013 despite significant investments in prevention programs [31]. More recent estimates from Samoa (2016) show a 20–40% decrease of adult suicide mortality attributed largely to the success in a ban on lethal pesticides like paraquat [4, 32–35]. Administering another GSHS in Samoa would be of great use to determine the possibility of similar success among adolescents.

Within our study, after adjusting for covariates, bullying remains a significant risk indicator associated with suicide attempt in both males and females. Our findings are consistent with the HBSC study, which found adolescents from Israel, Lithuania and Luxembourg who experienced cyberbullying and school bullying, had a significantly higher risk associated with suicidal ideations, plans and attempts [16]. From a LMIC perspective, the Young Lives study analyzed longitudinal predictors and associations of bullying in nearly 12,000 adolescents, over a 15-year period in Peru, Ethiopia, India and Vietnam through mixed methods. Their results suggest that bullying is corrosive and associated with long-term negative effects on self-esteem, self-efficacy, peer and parental relations [36]. The authors noted that indirect bullying (i.e. humiliation and social exclusion) was the most prevalent type of bullying experienced by age 15, in three of four countries ranging from 15% in Ethiopia to 28% in India. Our findings are also consistent with two recent systematic reviews which found that bullying perpetration and victimization via traditional (face-to-face) or cyberbullying were associated with deliberate self-harm in adolescents [37, 38].

Furthermore, our findings indicate a vulnerability to bullying during early adolescence suggesting early, preventive and context-appropriate interventions may be necessary to impact suicide behaviours and to help resolve mental health issues. Particularly, the results from GSHS corroborate this need in LMIC [39]. In fact, a previous systematic review and meta-analysis reviewed 99 studies on youth suicide interventions, where only 2% were conducted in a LMIC. Several challenges exist in these settings, including the paucity of mental health services and personnel, as well as, poor monitoring and evaluation systems [40]. Though school-based interventions have been effective in promoting mental health through education, there is potential to miss vulnerable adolescents, as dropout rates are higher in LMIC, especially in females, as compared to HIC [39]. Moreover, as bullying and cyberbullying have become a systemic public health issue, innovative approaches that transcend the school, including the integration of community, parental and mobile health interventions, are necessary to intervene early and prevent social marginalization and victimization. Given our findings, future research should focus on the generalizability of HIC bullying prevention and intervention models towards LMIC, as well as, understanding the temporality and progression of suicide risk indicators to ideation and suicide attempts in adolescents.

Our findings have important implications for policy and programmatic action. Notably, that adolescents are vulnerable humans who are susceptible to both positive and negative influences of environment, is evident from our work. Intervening with these individuals at critical points of entry, such as in schools, in families and within communities, is critical to bringing about sustainable change. To this end, governments should prioritize school-based intervention models to target both in-person and cyber bullying. Peer-to-peer support or self-help groups coordinated by teachers and administrators may be one approach to consider. Encouraging social activities that focus on building relationships and fostering a sense of trust, may be ways that schools and communities can prevent bullying and help adolescents build close friendships. Government’s focus on identifying and providing physical, emotional and social support to youth who have experienced injuries is important. For instance, through health walk-in clinics or through youth networks, would yield notable dividends on health and survival of this population. Widespread education campaigns on mental health and well-being for parents, teachers, public service offices and communities will be invaluable to building a supportive environment for at-risk youth.

Several limitations of this work should be considered. The GSHS’s cross-sectional design precludes temporal and causal inference. As the survey is school-based, information on important national, community and household indicators factors were missing, such as socioeconomic status, food security, family risk and protective factors, cultural and religious factors or national political climate. Additionally, in some LMIC where school attendance is low (especially among girls), the data may not be nationally representative. The GSHS did not ask specific questions on previous childhood abuse, behaviour, family history, previous mental illness or questions related to severity of depression which are also known risk indicators for poor mental health. Such data should be evaluated for inclusion in future phases of the GSHS. The sensitive nature of questions, self-reporting may have introduced bias due to under- or over-reporting on certain questions. Specific questions related to serious injury and bullying included in our analyses are limited in that they potentially under- or over- capture true responses. More specifically, ‘serious injury’ captures injuries from both intentional (violence, self-harm) and unintentional (road accidents) causes, while bullying may have only captured classical bullying (face-to-face), as opposed to the inclusion of cyberbullying. Sexual orientation may be a hidden risk indicator since it is not available in the current GSHS data. A recent systematic review reported higher rates of depression among young lesbian, gay, bisexual, transgender and questioning (LGBTQ) and LGBTQ-factors were associated with suicide risk [41, 42]. This may help explain some of the findings regarding bullying, injuries, etc. Since our analyses were ecological (i.e. using country-year data points), caution should be applied when making individual-level inferences. Lastly, since the GSHS design is intended to capture a representative sample of younger adolescents, prevalence estimates of older adolescents (16–17 years) may not be representative. While our results should be interpreted in light of these limitations, this pooled analysis represents a large number of participants in predominately LMIC, where much data on adolescents are lacking. Strengths of this analysis lie with the use of a validated tool administered in a large number of countries over approximately 15 years.

## Conclusion

Mental health has been recognized as an important priority in several global agendas, including Goal 3 of the Sustainable Development Goals and the WHO Comprehensive Mental Health Action Plan 2013–2020. Understanding the distribution and determinants of mental health-related conditions in the leaders of tomorrow – adolescents – is a critical step towards these goals. Programs should focus on improving social relationships throughout adolescence by sex, with specific themed interventions for younger and older adolescents. Our work should be used to continue and expand on the mental health dialogue and action worldwide.

## Supplementary information

**Additional file 1 Supplementary Fig. 1**. Country Prevalence of Students who Reported One or More Suicide Attempts in the Past 12 Months by WB Income Group by WHO Region. **Supplementary Fig. 2**. Country Prevalence of Reported Suicide Ideation in the Past 12 Months by WB Income Group by WHO Region. **Supplementary Fig. 3**. Pooled Prevalence Estimates per GSHS Question for HIC and LMIC, boys 13–15 Years. **Supplementary Fig. 4**. Pooled Prevalence Estimates per GSHS Question for HIC and LMIC, boys 16–17 Years. **Supplementary Fig. 5**. Pooled Prevalence Estimates per GSHS Question for HIC and LMIC, girls 13–15 Years. **Supplementary Fig. 6**. Pooled Prevalence Estimates per GSHS Question for HIC and LMIC, girls 16-17 Years. **Supplementary Fig. 7**2. Sensitivity Analysis. Mean Prevalence Estimates per GSHS Question, per phase, boys 13–15 Years. **Supplementary Fig. 8**. Sensitivity Analysis. Mean Prevalence Estimates per GSHS Question, per phase, girls 13–15 Years.

## Data Availability

Data are publicly available. The Global Student-based School Health Survey data that support the findings of this study are available in the CDC-GSHS repository at https://www.cdc.gov/gshs/countries/index.htm

## Abbreviations

ANOVA: Analysis of variance
CDC: Centers for Disease Control
GBD: Global Burden of Disease
GSHS: Global School-based Student Health Survey
HBSC: Health Behavior in School-aged Children
HIC: High-Income Countries
KYRBS: Korean Youth Risk Behavior Web-based Survey
LMIC: Low and middle-income countries
WB: World Bank
WHO: World Health Organization
YRBSS: Youth Risk Behavior Surveillance System
