# Supplementary material for: Suicidal behaviours among adolescents from 90 countries: a pooled analysis of the global school-based student health survey
Source: BMC Public Health. 2020 Aug 10;20:1102. doi: 10.1186/s12889-020-09209-z (PMC7416394; doi:10.1186/s12889-020-09209-z)
Supplement: Supplementary file 1 — Additional file 1 Supplementary Fig. 1. Country Prevalence of Students who Reported One or More Suicide Attempts in the Past 12 Months by WB Income Group by WHO Region. Supplementary Fig. 2. Country Prevalence of Reported Suicide Ideation in the Past 12 Months by WB Income Group by WHO Region. Supplementary Fig. 3. Pooled Prevalence Estimates per GSHS Question for HIC and LMIC, boys 13–15 Years. Supplementary Fig. 4. Pooled Prevalence Estimates per GSHS Question for HIC and LMIC, boys 16–17 Years. Supplementary Fig. 5. Pooled Prevalence Estimates per GSHS Question for HIC and LMIC, girls 13–15 Years. Supplementary Fig. 6. Pooled Prevalence Estimates per GSHS Question for HIC and LMIC, girls 16-17 Years. Supplementary Fig. 72. Sensitivity Analysis. Mean Prevalence Estimates per GSHS Question, per phase, boys 13–15 Years. Supplementary Fig. 8. Sensitivity Analysis. Mean Prevalence Estimates per GSHS Question, per phase, girls 13–15 Years. [file 12889_2020_9209_MOESM1_ESM.docx]

Supplementary Figure 1. Country Prevalence of Students who Reported One or More Suicide Attempts in the Past 12 Months by WB Income Group by WHO Region


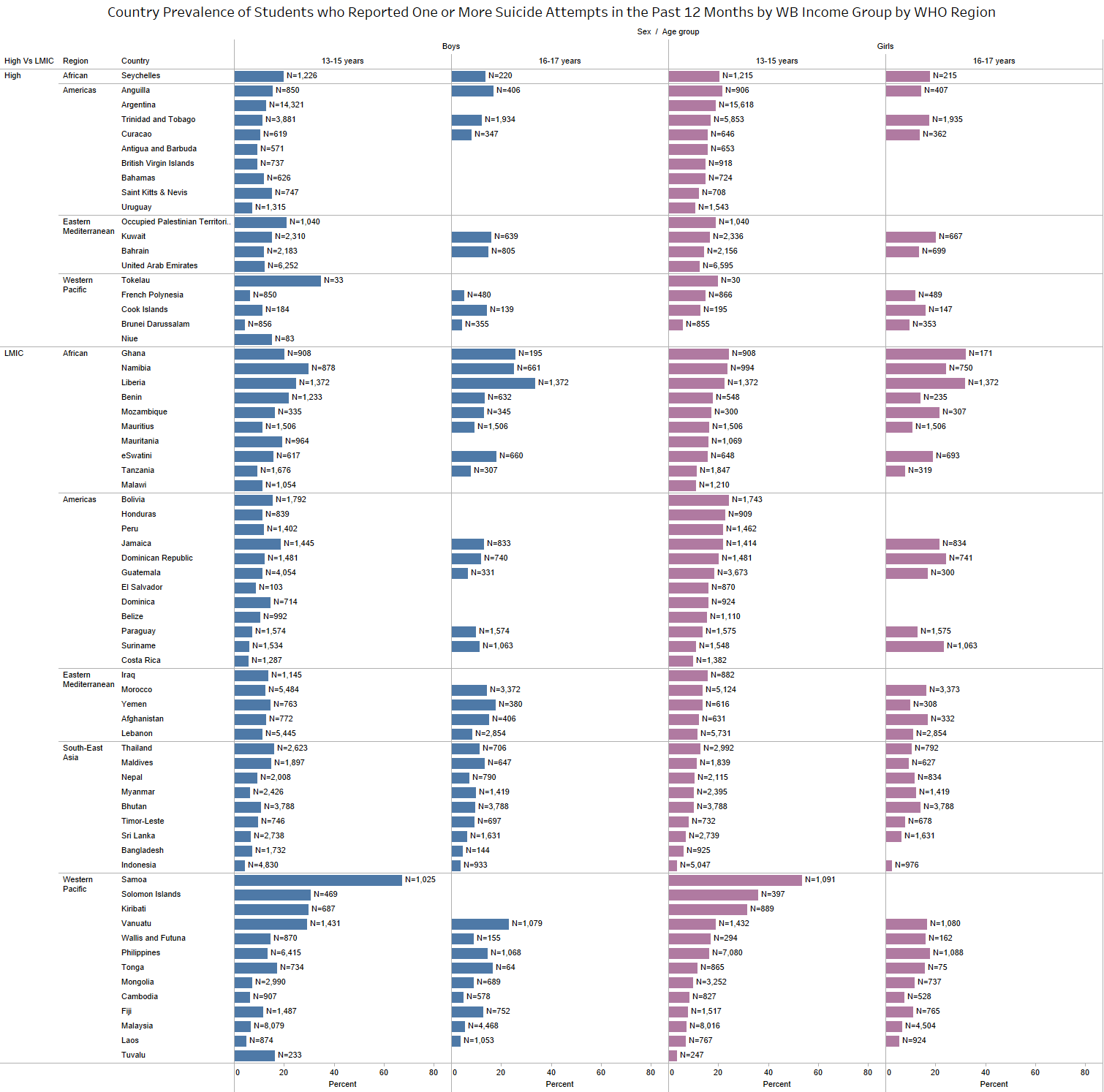


Supplementary Figure 2. Country Prevalence of Reported Suicide Ideation in the Past 12 Months by WB Income Group by WHO Region


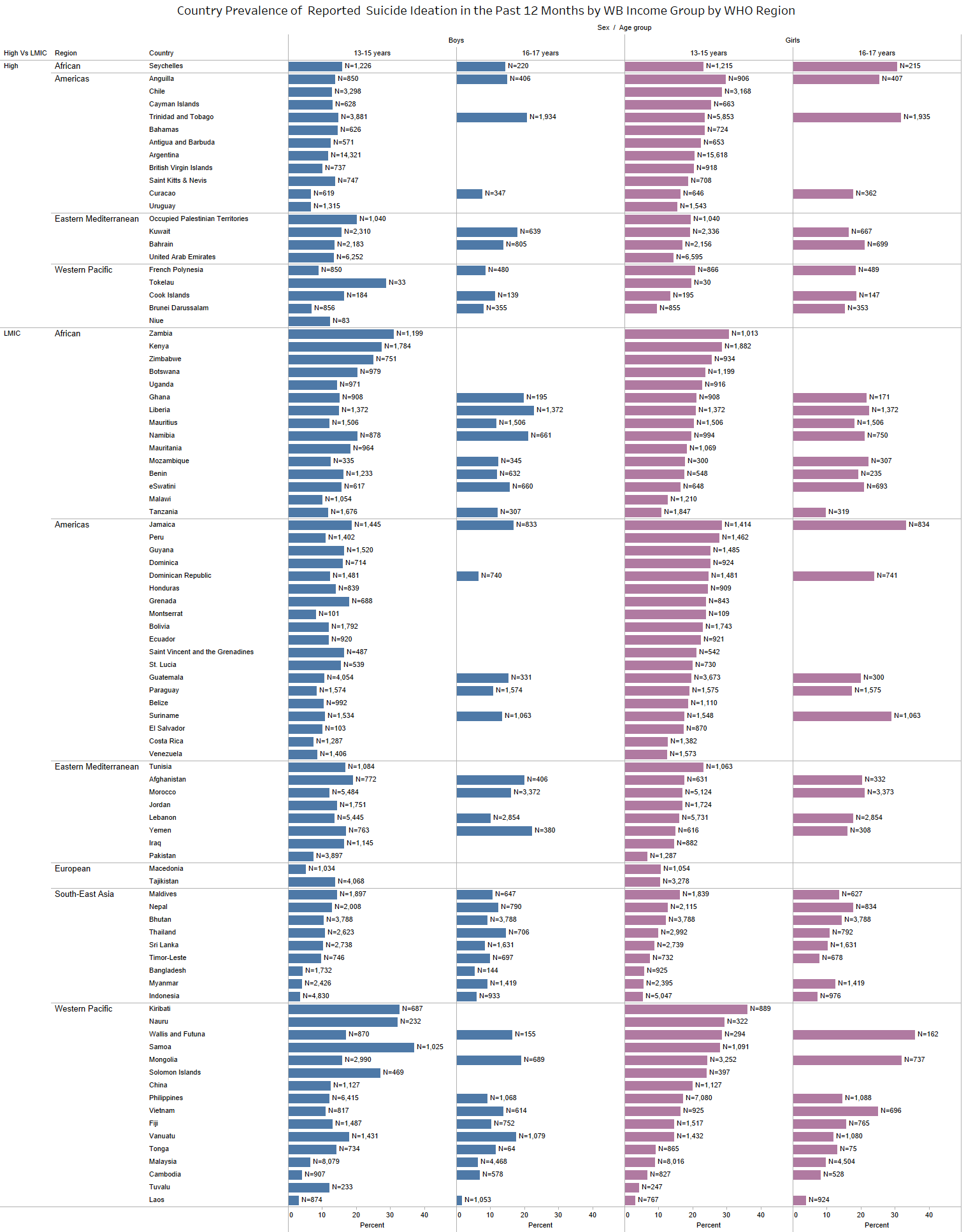


Supplementary Figure 3. Pooled Prevalence Estimates per GSHS Question for HIC and LMIC, boys 13-15 Years

Supplementary Figure 4. Pooled Prevalence Estimates per GSHS Question for HIC and LMIC, boys 16-17 Years

Supplementary Figure 5. Pooled Prevalence Estimates per GSHS Question for HIC and LMIC, girls 13-15 Years

Supplementary Figure 6. Pooled Prevalence Estimates per GSHS Question for HIC and LMIC, girls 16-17Years

Supplementary Figure 72. Sensitivity Analysis. Mean Prevalence Estimates per GSHS Question, per phase, boys 13-15 Years

Supplementary Figure 8. Sensitivity Analysis. Mean Prevalence Estimates per GSHS Question, per phase, girls 13-15 Years

Figure 8. Sensitivity Analysis. Mean Prevalence Estimates per GSHS Question for adolescents 13-15 years
